# The cellular chloride channels CLIC1 and CLIC4 contribute to virus-mediated cell motility

**DOI:** 10.1074/jbc.RA117.001343

**Published:** 2018-02-08

**Authors:** Gabrielė Stakaitytė, Nnenna Nwogu, Jonathan D. Lippiat, G. Eric Blair, Krzysztof Poterlowicz, James R. Boyne, Andrew Macdonald, Jamel Mankouri, Adrian Whitehouse

**Affiliations:** From the ‡ School of Molecular and Cellular Biology,; § Astbury Centre for Structural Molecular Biology,; ¶ School of Biomedical Sciences, Faculty of Biological Sciences, University of Leeds, Leeds, LS2 9JT, United Kingdom and; ‖ Centre for Skin Sciences, School of Chemistry and Biosciences, Faculty of Life Sciences, University of Bradford, Bradford BD7 1DP, United Kingdom

**Keywords:** cell motility, cell migration, channel activation, chloride channel, viral protein

## Abstract

Ion channels regulate many aspects of cell physiology, including cell proliferation, motility, and migration, and aberrant expression and activity of ion channels is associated with various stages of tumor development, with K^+^ and Cl^−^ channels now being considered the most active during tumorigenesis. Accordingly, emerging *in vitro* and preclinical studies have revealed that pharmacological manipulation of ion channel activity offers protection against several cancers. Merkel cell polyomavirus (MCPyV) is a major cause of Merkel cell carcinoma (MCC), primarily because of the expression of two early regulatory proteins termed small and large tumor antigens (ST and LT, respectively). Several molecular mechanisms have been attributed to MCPyV-mediated cancer formation but, thus far, no studies have investigated any potential link to cellular ion channels. Here we demonstrate that Cl^−^ channel modulation can reduce MCPyV ST-induced cell motility and invasiveness. Proteomic analysis revealed that MCPyV ST up-regulates two Cl^−^ channels, CLIC1 and CLIC4, which when silenced, inhibit MCPyV ST-induced motility and invasiveness, implicating their function as critical to MCPyV-induced metastatic processes. Consistent with these data, we confirmed that CLIC1 and CLIC4 are up-regulated in primary MCPyV-positive MCC patient samples. We therefore, for the first time, implicate cellular ion channels as a key host cell factor contributing to virus-mediated cellular transformation. Given the intense interest in ion channel modulating drugs for human disease. This highlights CLIC1 and CLIC4 activity as potential targets for MCPyV-induced MCC.

## Introduction

During virus-induced transformation, alterations in the biological properties of infected cells by viral oncogenes confers properties that can drive tumor progression (1). The subversion of several host pathways has been linked to the oncogenic ability of viral proteins, but the modulation of host cell ion channel activity and the contribution of this important virus–host interaction to viral oncogenesis has not been studied. Ion channels regulate many aspects of cell physiology, including cell proliferation, motility, and migration, making them an attractive target for viral manipulation (2, 3). The aberrant expression and activity of ion channels is associated with various stages of tumor development, and emerging *in vitro* and preclinical studies have revealed that pharmacological manipulation of ion channel activity offers protection against several cancers (4). Merkel cell carcinoma (MCC)^4^ is a highly aggressive neuroendocrine carcinoma of the skin. MCC is characterized by the appearance of local or regional lymph nodes with a high propensity for distant metastases in various sites (5). Accordingly, MCC has a poor 5-year survival rate. The reported cases of MCC have tripled in the last 20 years in both Europe and the United States, because of an increase in associated risk factors, including UV exposure, immunosuppression, and increased age (6).

In 2008, Merkel cell polyomavirus (MCPyV) was identified to be clonally integrated in ∼80% of MCC tumors (7). Like other polyomaviruses, MCPyV expresses a variety of early spliced variant regulatory proteins required for viral replication and pathogenesis, including the small and large tumor antigens (ST and LT, respectively) (8). MCPyV infection and integration appear to occur prior to tumor cell expansion, with additional truncated LT mutations observed which render MCPyV replication defective (7). Notably, depletion studies have demonstrated that both ST and LT protein are required for MCC survival and proliferation (9). However, in contrast to other polyomaviruses, MCPyV ST is sufficient to transform rodent cells to anchorage- and contact-independent growth and also induces serum-free proliferation of human cells (10). Recent studies have explored the contribution of MCPyV ST to MCC development and proliferation, suggesting it is highly multifunctional. MCPyV ST functions as an inhibitor of NF-κB–mediated transcription (11) and leads to the hyperphosphorylation of the translation regulatory protein, 4E-BP1, resulting in dysregulation of cap-dependent translation (10). It also promotes transcriptional changes in glycolytic metabolic pathways (12). Moreover, MCPyV ST prevents SCF^Fwb7^-mediated degradation of MCPyV LT and several cellular oncoproteins (13). MCPyV ST also promotes differential expression of cellular proteins involved in microtubule and actin-associated cytoskeletal organization and dynamics, which leads to a motile and migratory phenotype (14, 15). Notably, this MCPyV ST-induced migratory phenotype may be associated with the highly metastatic nature of MCC tumors. This is also supported by recent studies showing that engraftment of MCC cell lines into SCID mice results in circulating tumor cells and metastasis formation (16).

We demonstrate herein that two members of the chloride channel family, a diverse group of anion selective channels, are up-regulated by MCPyV ST to induce motility and invasiveness during MCPyV-induced metastasis. This for the first time implicates cellular ion channels as a contributing factor to virus-mediated cellular transformation.

## Results

### Cl^−^ channel modulators inhibit MCPyV ST-induced cell motility

**Figure 1.**
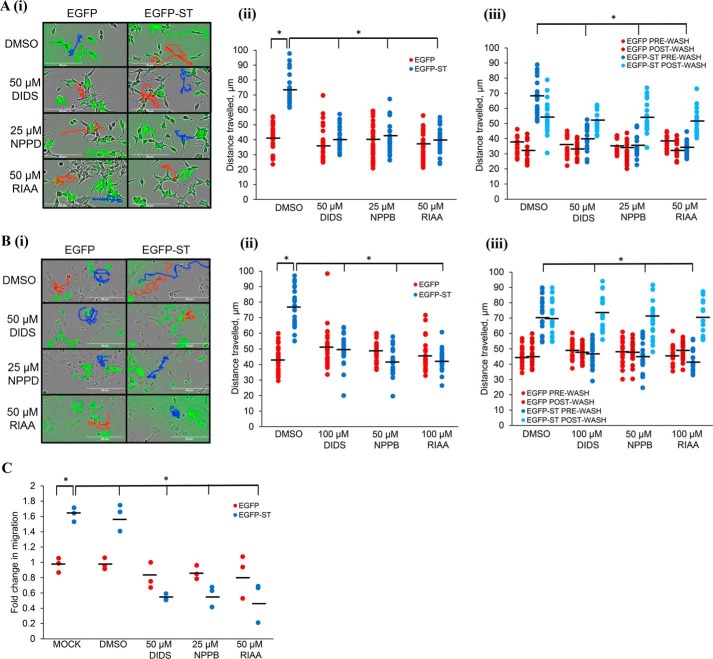
**Cl^−^ channel blockers inhibit the metastatic phenotypic changes associated with ST expression.** *A* and *B*, in (*A*) i293-EGFP and i293-EGFP-ST cells were induced with doxycycline hyclate and in (*B*) MCC13 cells were transfected with EGFP and EGFP-ST. (*i*) cells were then treated with DMSO, DIDS, RIAA, or NPPB and after 24 h, cell motility was measured using the IncuCyte kinetic imaging system. Images were taken every 30 min for a 24-h period. (*ii*) scatter plot showing cell movement tracked using ImageJ (*n* = 25). Average cell movement, as indicated by the *horizontal bar*, was calculated and significance tested using a two-tailed Student's *t* test; *, *p* < 0.001. (*iii*) After 24 h, inhibitors were washed off cells and media added. Cells were imaged for a further 24 h and average movement calculated (*n* = 25); *, *p* < 0.001. *C*, i293-EGFP and i293-EGFP-ST cells were induced and treated with DMSO, DIDS, RIAA, or NPPB. After 24 h, cells were transferred into migration wells and inhibitors added at appropriate concentrations to allow cells to migrate from serum-free to 10% FBS conditions for 24 h. Migratory cells were stained and measured at 560 nm to quantify migration. Average cell migration was calculated and significance tested using a two-tailed Student's *t* test (*n* = 3); *, *p* < 0.001.

Viral proteins modulate cellular ion channel functionality to favor their life cycles (2). In recent years, Cl^−^ channels have emerged as some of the most active cellular ion channels during tumorigenesis, because of their involvement in both cell motility and proliferation (17, 18). We reasoned that Cl^−^ channels could therefore play a role in MCPyV ST-induced cell motility. If so, Cl^−^ channel blockers, including diisothiocyanostilbene-2,20-disulfonic acid (DIDS), 5-nitro-2–3-phenylpropylamino benzoic acid (NPPB), and R(+)-indyanyloxyacetic acid 94 (RIAA) (3) would be predicted to affect MCPyV ST-induced cell motility. This was investigated in two independent cell lines under conditions of MCPyV ST expression. i293-EGFP and i293-EGFP-ST cells were induced using doxycycline hyclate (14), whereas MCC13 cells (an MCPyV-negative Merkel carcinoma cell line) were transfected with enhanced GFP (EGFP) or EGFP-ST, and treated with each respective inhibitor for 24 h. Cell motility was assessed through IncuCyte imaging, every 30 min for 24 h and the distance traveled by individual cells tracked using ImageJ. Fig. 1, *A* and *B* shows that MCPyV ST-expressing cells displayed larger average distances traveled (1.7-fold increase in motility in ST-expressing cells, *n* = 25, *p* < 0.001) indicative of enhanced cell motility in both cell lines, consistent with previous findings (14). Fig. 1, *A* and *B* also demonstrates that, upon the addition of DIDS, NPPB, and RIAA, no significant differences were observed between the average distances traveled by control GFP-expressing cells, but the motility of MCPyV ST-expressing cells significantly decreased following drug treatments in both cell types (reduced to baseline motility, *n* = 25, *p* < 0.001). The effects of the drugs were not additive when combined, and validated as nontoxic at the concentrations assessed by MTS assay (Fig. S1). The effects of DIDS, NPPB, and RIAA were reversible, as when the drugs were washed from the cells for 24 h, a significant recovery of cell motility was observed most notably in MCPyV ST expressing MCC13 cells (Fig. 1, *A* and *B*(*iii*), *n* = 25, *p* < 0.001).

To further confirm these effects, haptotaxis migration assays were performed in the presence of DIDS, NPPB, and RIAA. This assay investigates the three-dimensional migration of cells toward a chemoattractant across a permeable chamber. Induced i293-EGFP and i293-EGFP-ST cells were transferred to haptotaxis plates and treated with DIDS, NPPB, and RIAA. Cells were allowed to migrate for 24 h and migration was assessed by immunofluorescent staining of cells that had migrated into the chambers. Fig. 1*C* shows that i293-EGFP-ST cells displayed enhanced migration compared with control (*n* = 3, *p* < 0.001), which was significantly reduced by treatment with DIDS, NPPB, and RIAA, suggesting that MCPyV ST-induced migration is similarly Cl^−^ channel dependent. Together these data suggest that cellular Cl^−^ channels are involved in MCPyV ST-induced cell motility and migration, and thus may contribute to MCPyV ST-induced metastatic processes.

### MCPyV ST enhances the expression of CLIC1 and CLIC4

**Figure 2.**
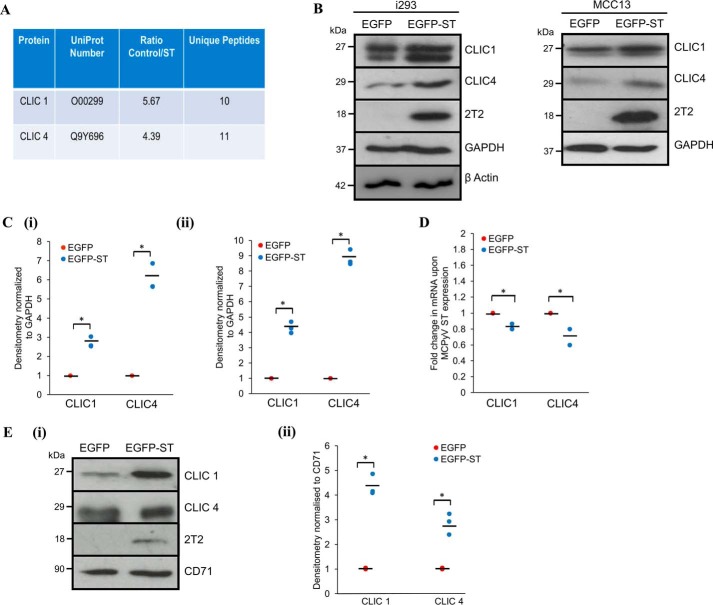
**MCPyV ST up-regulates CLIC proteins.** *A*, quantitative proteomic analysis shows an increase in CLIC family protein levels upon MCPyV ST expression. *B*, i293-EGFP or i293-EGFPST cells were incubated for 48 h in the presence of doxycycline, whereas MCC13 cells were transfected with EGFP or EGFP-ST for 24 h. Cell lysates were probed using CLIC1- and CLIC4-specific antibodies. GAPDH and β actin were used to ensure equal loading. The 2T2 hybridoma was used to probe for MCPyV ST expression. *C*, densitometry was performed using ImageJ (*n* = 3) for (*i*) i293 or (*ii*) MCC13 samples. *, *p* < 0.01. *D*, total RNA was extracted from induced i293-EGFP or i293-EGFP-ST cells, reverse transcribed, and RT-quantitative PCR performed. Transcript levels were analyzed using the comparative CT method (*n* = 3). *, *p* < 0.01. *E*, (*i*) surface biotinylation experiments were performed in induced i293-EGFP or i293-EGFP-ST cells; lysates were probed for CLIC1, CLIC4, and CD71 as a surface marker control. (*ii*) densitometry of Western blots was performed using ImageJ software (*n* = 3). *, *p* < 0.01.

We next investigated the identity of the Cl^−^ channel(s) required by MCPyV ST to enhance cell motility and migration. Approximately 40 genes encode proteins classified as Cl^−^intracellular-channel (CLIC) proteins, cyclic AMP–gated (CFTR), calcium-activated (CaCC), voltage-activated Cl^−^ channels and Cl^−^/H^+^ exchangers (CLCs), as well as ligand-gated Cl^−^ channels (GABA_A_, GABA_C_, and glycine) (19). We re-analyzed our previous quantitative proteomic datasets to determine potential alterations in the host cell channelome on inducible MCPyV ST expression (14). Analysis revealed that MCPyV ST expression up-regulates the expression of two CLIC family proteins CLIC1 (Fig. 2*A*, 5.67-fold increase of ST/control cells) and CLIC4 (Fig. 2*A*, 4.39-fold increase of ST/control cells). To confirm these datasets, we assessed the levels of CLIC1 and CLIC4 expression upon MCPyV ST induction by immunoblotting cell lysates obtained from induced i293-EGFP and i293-EGFP-ST cells and MCC13 cells transfected with EGFP or EGFP-ST for 48 h. Fig. 2*B* shows that both CLIC1 and CLIC4 were up-regulated upon MCPyV ST expression in both cell lines, confirming their up-regulation (Fig. 2, *B* and *C*). This increase in CLIC1/CLIC4 levels occurred at the protein level, as transcription levels assessed by RT-quantitative PCR remained relatively unchanged in both cell types (Fig. 2*D*). The increase in expression was reflected by an increase in the cell surface expression of CLIC1 and CLIC4 in i293-EGFP-ST cells compared with EGFP control, assessed using surface biotinylation assays (Fig. 2*E*(*i* and *ii*)). This was not a global effect on cell surface protein expression levels because the levels of transferrin receptor (CD71) were unaffected by MCPyV ST (Fig. 2*E*(*i* and *ii*)). Together these data confirm that CLIC1 and CLIC4 are up-regulated upon MCPyV ST expression.

### Depletion of CLIC1 and CLIC4 disrupts MCPyV ST-induced cell motility

**Figure 3.**
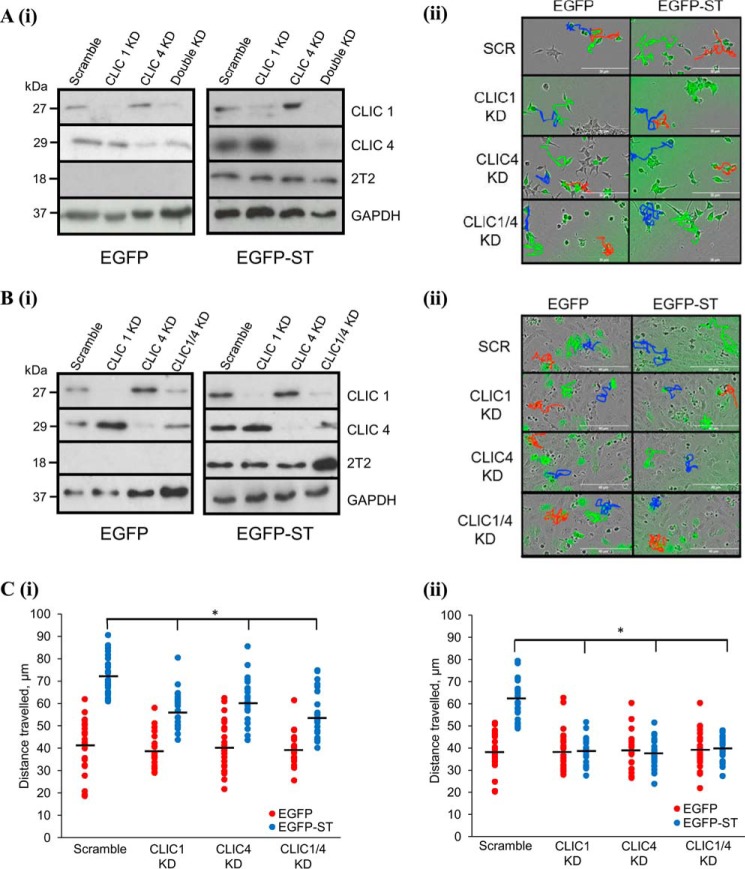
**CLIC1 and CLIC4 KD inhibits MCPyV ST-induced cell motility.** *A* and *B*, in *A*(*i*) i293-EGFP and i293-EGFP-ST cell lysates or (*B*)(*i*) EGFP or EGFP-ST-transfected MCC13 cells were probed for successful knockdown with CLIC1- and CLIC4-specific antibodies. GAPDH was used as loading control and 2T2 hybridoma was used to show MCPyV ST expression. *A*(*ii*), i293-EGFP and i293-EGFP-ST cells were transfected with scrambled or CLIC1 and/or CLIC4 siRNA for 12 h, then induced using doxycycline for 24 h. *B*(*ii*), MCC13 cells were transfected with scrambled or CLIC1 and/or CLIC4 siRNA for 12 h, then retransfected with EGFP and EGFP-ST after a further 12 h. 48 h post knockdown, cell motility was measured using the IncuCyte kinetic imaging system. Images were taken every 30 min for a 24-h period. *C*, scatter plots showing cell movement of (*i*) i293 or (*ii*) MCC13 cells tracked using ImageJ (*n* = 25). Average cell movement, indicated by *horizontal bar*, was calculated and significance was tested using a two-tailed Student's *t* test. *, *p* < 0.001.

**Figure 4.**
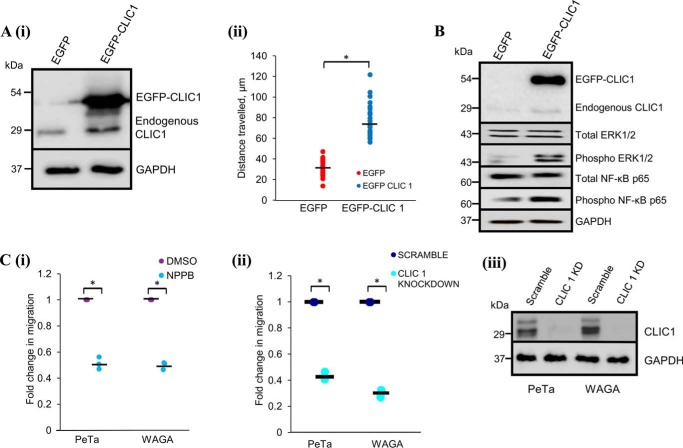
**CLIC1 overexpression is required to induce cell motility in 293 and MCPyV-positive MCC cell lines.** *A*, 293 cells were transfected with EGFP or EGFP-CLIC1 for 24 h. (*i*) cell lysates were probed using CLIC1-specific antibody; GAPDH was used to ensure equal loading. (*ii*) scatter plot showing cell movement tracked using ImageJ (*n* = 25). Average cell movement, as indicated by the *horizontal bar*, was calculated and significance tested using a two-tailed Student's *t* test. *, *p* < 0.001. *B*, 293 cells were transfected with EGFP or EGFP-CLIC1 for 24 h. Cell lysates were probed using CLIC1, ERK1/2, and NF-κB–specific antibodies; GAPDH was used to ensure equal loading. *C*, MCPyV-positive MCC cell lines, PeTa, and WAGA were (*i*) incubated with DMSO or NPPB or (*ii*) transfected with scrambled or CLIC1 siRNA for 12 h. Cells were then transferred into migration wells and allowed to migrate from serum-free to 10% FBS conditions for 24 h. Migratory cells were stained and measured at 560 nm to quantify migration. Average cell migration was calculated and significance tested using a two-tailed Student's *t* test (*n* = 3). *, *p* < 0.001. (*iii*) PeTa and WAGA cells were probed to confirm successful knockdown with CLIC1-specific antibodies. GAPDH was used as loading control.

Because of the increased expression of CLIC1 and CLIC4 in MCPyV ST-expressing cell lines, we investigated the effects of CLIC1/CLIC4 silencing on MCPyV ST-induced cell motility. Induced i293-EGFP and i293-EGFP-ST cells or MCC13 cells transfected with EGFP or EGFP-ST were transfected with CLIC1- and/or CLIC4-specific siRNA. CLIC1/CLIC4 depletion both as single and double knockdowns was confirmed via immunoblotting (Fig. 3, *A* (*i*) and *B*(*i*)). When the motility of MCPyV ST-expressing i293 and MCC13 cells was assessed in CLIC1/CLIC4 silenced cells (IncuCyte kinetic, cells imaged every 30 min for 24 h), significant decreases in the average distances traveled were observed compared with scrambled siRNA controls, most notably in MCPyV ST MCC13 cells (return to baseline motility, *n* = 25, *p* < 0.001) (Fig. 3, *A* (*ii*) and *B*(*ii*)). Interestingly, differences in the average distance traveled for non-MCPyV ST-expressing cells following knockdown of CLIC1 and/or CLIC4 were not observed, suggesting they only contribute to the enhanced motility observed following MCPyV ST expression (Fig. 3, *A–C*) (*n* = 25). To confirm that the enhanced motility observed in MCPyV ST-expressing cells is because of CLIC1/4 overexpression we performed gain-of-function experiments to assess the effect of CLIC1 overexpression in non-MCPyV ST-expressing 293 cells. Motility of EGFP-CLIC1–expressing cells (IncuCyte kinetic, cells imaged every 30 min for 24 h) displayed a significant increase in cell motility over GFP control cells (Fig. 4*A*) (*n* = 25, *p* < 0.001) consistent with CLIC1 induction leading to an enhanced motile phenotype. We also observed enhanced phosphorylation of ERK1/2 and NF-κB p65 subunit following CLIC1 overexpression (Fig. 4*B*), the activation of which has been shown to enhance motility in a range of cancers (20, 21). Activation of these signaling pathways may therefore contribute to the metastatic phenotype observed following CLIC1 overexpression.

Furthermore, to demonstrate that the enhanced Cl^−^ channel activity is required for cell motility and migration of MCPyV-positive MCC cell lines, haptotaxis migration assays were performed in the presence of NPPB at nontoxic concentrations assessed by MTS assay (Fig. S2), or upon siRNA-mediated CLIC1 depletion, using two MCPyV-positive MCC cell lines. Cells were allowed to migrate for 24 h and migration was assessed by immunofluorescent staining of cells that had migrated into the chambers. Fig. 4*C* shows that migration of MCPyV-positive MCC cell lines were significantly reduced compared with control (*n* = 3, *p* < 0.001), upon treatment with either NPPB or CLIC1 depletion, suggesting that MCPyV-positive MCC cell line migration is similarly Cl^−^ channel dependent. Together these data demonstrate that CLIC1 or CLIC4 depletion specifically inhibits MCPyV ST–induced motility, suggesting that these Cl^−^ channels mediate the effects observed for pharmacological Cl^−^ channel modulation.

### MCPyV-positive MCC tumors overexpress CLIC1 and CLIC4

**Figure 5.**
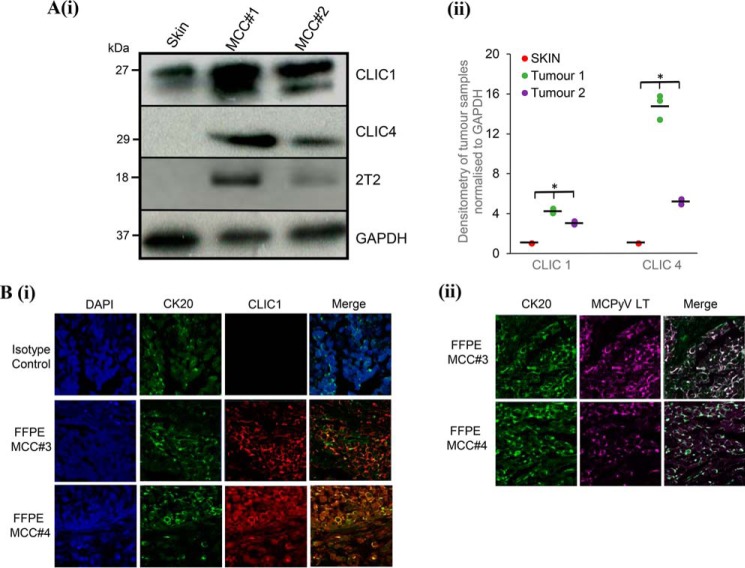
**CLIC1 and CLIC4 are up-regulated in MCC.** *A* (*i*), healthy skin and two independent MCC biopsy samples (MCC#1 and MCC#2) were crushed using a mortar and pestle on dry ice, lysed with RIPA buffer and then further homogenized by sonication. Tissue lysates were probed for CLIC1, CLIC4, GAPDH, and 2T2. (*ii*) densitometry of immunoblots was performed using ImageJ (*n* = 3). *, *p* < 0.001. *B*, formalin-fixed, paraffin-embedded (FFPE) sections from primary MCC tumors (MCC#3 and MCC#4) were probed for (*i*) CK20 and CLIC1 and (*ii*) CK20 and MCPyV LT to demonstrate MCC sections were MCPyV positive. An isotype-matched antibody was used as a negative control in parallel. Nuclei were counterstained with DAPI. Representative images captured with a Zeiss LSM 510 confocal microscope are shown.

Finally, we assessed whether CLIC1 and CLIC4 are expressed and/or up-regulated in MCC tumors. Immunoblotting analysis was performed on cellular lysates of two independent MCPyV-positive MCC tumor samples (MCC#1 and #2) comparing protein levels against a negative non-tumor cadaveric skin sample. Fig. 5*A*(*i*) and resulting densitometry analysis (Fig. 5*A*(*ii*)) show that CLIC1 and CLIC4 expression is enhanced in both MCC samples compared with healthy skin. To further investigate the differential expression of CLIC proteins in the context of MCC, multicolor immunochemistry analysis was performed on formalin-fixed, paraffin-embedded (FFPE) sections of primary MCPyV-positive MCC tumors distinct from the tumor samples used above (MCC#3 and #4). Sections were incubated with antibodies specific for CLIC1 and cytokeratin 20 (CK20), a marker widely used to distinguish MCC and MCPyV LT (Fig. 5*B*). An isotype-matched control was also used as a negative control. Results confirmed increased levels of CLIC1 coincident with CK20 in regions of the tumor.

Finally, to determine whether CLIC1 and CLIC4 are significantly increased in MCPyV-positive MCC compared with MCPyV-negative MCC, gene expression profiles for a total of 94 patients were obtained from a publicly available dataset (accession number GSE39612 (22)). Expression profiles were preprocessed, including background correction, normalization, and summation of the intensities for each sample using R/Bioconductor (23) and the R limma package used to call differentially expressed genes (24). Strikingly, we identified a significant increase in CLIC1 (2.5-fold, *p* = 0.02) and CLIC4 (3.5-fold, *p* = 0.002) in MCPyV-positive MCC compared with MCPyV-negative MCC control samples. Moreover, CLIC1 expression was also significantly increased (2.3-fold, *p* = 0.04) in metastatic MCPyV-positive MCC compared with a metastatic MCPyV-negative MCC control, suggesting a link between MCC MCPyV status and progression. Intriguingly, several other chloride ion channels from the CLIC and CLCN families were also dysregulated in a MCPyV-specific manner. Together, these data support our *in vitro* observations and suggest a link between MCPyV-mediated CLIC up-regulation and MCC pathogenesis. Overall, these results confirm that CLIC1 and CLIC4 are up-regulated during MCPyV infection *in vivo*, implicating a novel role for these channels in MCPyV-positive MCC tumorigenesis.

## Discussion

Growing evidence supports a role of cellular ion channels in cancer development, but somewhat surprisingly, their contribution to virus-induced cancer has yet to be documented. Cl^−^ channels are considered one of the most active channel families during neoplastic transformation as they control ion homeostasis, pH levels, and cell volume, with the latter being particularly important for cancer cell migration and infiltration. We sought to assess the potential contribution of Cl^−^ channels to the metastatic processes that occur during MCPyV infection. We show that MCPyV ST enhances the expression of CLIC1 and CLIC4 in transformed cell lines and primary MCC samples. When these channels are silenced or pharmacologically inhibited, MCPyV ST-induced motility is reduced. This, for the first time, identifies cellular Cl^−^ channels that directly contribute to the metastatic processes of a viral oncogene.

The dependence on CLIC1/CLIC4 during MCPyV ST metastatic processes opens new insights into how viruses manipulate susceptible cells to mediate cancer progression. DNA viruses can encode viroporins (25), a group of viral ion channel proteins that participate in several viral functions, including the promotion of release of virus particles, modulation of cellular vesicles, and membrane permeability. MCPyV has no known viroporin, suggesting it is likely that MCPyV proteins interfere with the normal channelome to benefit virus replication. The ability of MCPyV ST to specifically up-regulate CLIC1/CLIC4 promotes metastatic activities in virus-infected cells. This is consistent with previous studies that identified the overexpression of CLIC1/CLIC4 in numerous cancer tissues including colon, prostate, pancreatic, and ovarian (26–28), suggesting they directly contribute to tumor development. Interestingly, MCPyV-positive tumors were recently shown to display elevated expression of *GABRB3* and K^+^ channel genes at the transcriptional level, which may play additional roles in the control of ionic balance during tumor growth (29).

A key question remains, how does MCPyV ST target CLIC1 and CLIC4, and how does this ultimately benefit the virus? Both channels are widely expressed and their activity regulates components of the cytoskeleton, adhesion molecules, and cell signaling pathways (18, 27, 30), all of which may contribute to MCPyV ST tumor formation. We observed that the MCPyV ST effects on CLIC1/CLIC4 did not occur at the transcriptional level, ruling out the involvement of known MCPyV ST cellular targets including p53 and tumor necrosis factor α (TNFα) (31–33) that regulate CLIC transcript levels. Others have established that inducible NOS, through regulating channel nitrosylation, can control the expression levels of CLICs through protein degradation (34). Although the ability of MCPyV ST to modulate inducible NOS has not been reported, it is currently under investigation. Moreover, it is not known whether the modified levels of CLIC1/CLIC4 mediate alterations in their intracellular activity in cellular organelles, as the analysis of channel functionality at these membranes is challenging. Thus, although it can be concluded that MCPyV ST-induced CLIC1/CLIC4 overexpression promotes a tumorigenic state, the mechanisms behind this regulation and their molecular contribution to MCC requires further investigation. It can be noted, however, that MCPyV ST most likely exploits CLIC1 and CLIC4 for a common cellular effect because simultaneous silencing of the channels resulted in no synergistic inhibition of MCPyV ST-induced cell motility. In addition, the observation that CLIC1 overexpression can activate ERK1/2 and NF-κB p65 raises the possibility that MCPyV ST targets CLIC1/CLIC4 to regulate the mitogenic signaling pathways that contribute to cancer metastasis (20, 21).

In conclusion, we reveal that MCPyV ST requires functional CLIC1 and CLIC4 channels to promote cell motility and invasion. To date, compounds specifically targeting CLIC1 and CLIC4 are not clinically available, and no CLIC1/CLIC4 blockers have advanced into clinical trials. However, with the emerging interest in this ion channel family as an anti-cancer target, inhibiting these channels may emerge as a novel therapeutic approach for the treatment of MCC.

## Experimental procedures

### Mammalian cell culture

Inducible HEK-293-ST Flip-In cells, described previously (11, 14), were maintained in Dulbecco's modified Eagle's medium (DMEM) containing 10% fetal bovine serum and 1% penicillin-streptomycin. MCC13, WAGA (9), and PeTa (35) cells were maintained in RPMI 1640 media supplemented with 10% FBS and 1% penicillin-streptomycin.

### Plasmids, siRNAs, and antibodies

EGFP-ST expression vectors have been described previously (11). EGFP-CLIC1 expression construct was purchased from OriGene. CLIC1- and CLIC4-specific siRNAs were purchased from Qiagen (product no. 1027416). CLIC1 and CLIC4 antibodies were purchased from Santa Cruz Biotechnology (sc-81873) and Abcam (ab76593), respectively. Antibodies were used at 1:5000 dilution. The 2T2 hybridoma was provided by Dr. Buck, National Cancer Institute, Bethesda, MD.

### Cl^−^ channel modulators

RIAA (Sigma-Aldrich), NPPB (Tocris Bioscience), and DIDS (Sigma-Aldrich) were used at the highest nontoxic concentrations: 50, 25, and 50 μm for HEK 293 cells, and 100, 50, 100 μm for MCC13 cells, respectively.

### Immunoblotting

Skin and MCC tumor biopsy samples were crushed using a pestle and mortar on dry ice and homogenized by sonication prior to lysis in RIPA (50 mm Tris-HCl pH 7.6, 150 mm NaCl, 1% Nonidet P-40), supplemented with protease inhibitor mixture (Roche) as described previously (36). Proteins were separated by SDS-PAGE, transferred to nitrocellulose membranes, and probed with the appropriate primary and HRP-conjugated secondary antibodies, as described previously (37).

### Live cell imaging

Cell motility was analyzed using IncuCyte kinetic live cell imaging (14). Imaging was performed for 24 h, with images taken every 30 min. Cell motility was tracked using ImageJ software.

### Haptotaxis migration assay

Migration assays were performed using CytoSelect 24-well Haptotaxis Assay Collagen I coated plates (Cell Biolabs, Inc.), as directed by the manufacturer.

### Quantitative RT-PCR

RNA was extracted using TRIzol (Invitrogen) and DNase treated as described previously (38). RNA concentration and quality were compared using a NanoDrop 1000 spectrophotometer (NanoDrop Technologies) at 280 nm. RNA (1 μg) from each fraction was reverse transcribed with SuperScript II (Invitrogen) using oligo(dT) primers (Promega). mRNA quantitative analysis was performed using the comparative CT method as described previously (39). Primers used were as follows: CLIC1 (forward), GAC ACC TTC CAT CTT CAG CAC T; CLIC1 (reverse), CAA GAA TTC AAA CCC AGC ACT C; CLIC4 (forward), CAT CCG TTT TGA CTT CAC TGT TG; CLIC4 (reverse), AGG AGT TGT ATT TAG TGT GAC GA.

### Cell viability assays

Cell viability was assessed 24 h post treatment using the CellTiter 96 AQueous One Solution Cell Proliferation Assay reagent (Promega) as described previously (40).

### Multicolor immunohistochemistry

Formalin-fixed, paraffin-embedded (FFPE) sections from primary MCC tumors were procured from OriGene and analyzed as described previously (41). Primary antibodies were anti-CK20 (Dako Products, dilution 1:50), anti-CLIC1 and -CLIC4 (Santa Cruz Biotechnology and Abcam, respectively, dilution 1:250), and anti-MCPyV LT (Santa Cruz Biotechnology, dilution 1:100). An isotype-matched irrelevant antibody was used as a negative control on serial sections of tissues in parallel.

## Author contributions

G. S., N. N., G. E. B., K. P., J. R. B., and J. M. data curation; G. S., N. N., J. D .L., K. P., J. R. B., A. M., and A. W. formal analysis; G. S., N. N., J. D. L., J. R. B., A. M., and J. M. investigation; J. D. L., A. M., J. M., and A. W. conceptualization; G. E. B. resources; G. E. B. and K. P. methodology; J. R. B., A. M., J. M., and A. W. writing-original draft; A. M. and A. W. supervision; J. M. and A. W. funding acquisition; J. M. and A. W. writing-review and editing; A. W. project administration.

## Supplementary Material

Supporting Information

## Footnotes

4 The abbreviations used are:

MCC: Merkel cell carcinoma
MCPyV: Merkel cell polyomavirus
ST: small tumor
LT: large tumor
DIDS: diisothiocyanostilbene-2,20-disulfonic acid
NPPB: 5-nitro-2–3-phenylpropylamino benzoic acid
FFPE: formalin-fixed, paraffin-embedded
EGFP: enhanced green fluorescent protein
MTS assay: 3-(4,5-dimethylthiazol-2-yl)-5-(3-carboxymethoxyphenyl)-2-(4-sulfophenyl)-2*H*-tetrazolium, inner salt
RIAA: R(+)-indyanyloxyacetic acid 94.
