# Supplementary material for: The cellular chloride channels CLIC1 and CLIC4 contribute to virus-mediated cell motility
Source: J Biol Chem. 2018 Feb 8;293(12):4582–90. doi: 10.1074/jbc.RA117.001343 (PMC5868249; doi:10.1074/jbc.RA117.001343)
Supplement: Supporting Information [file supp_293_12_4582__index.html]

The cellular chloride channels CLIC1 and CLIC4 contribute to virus-mediated cell motility — MCPyV ST enhances Cl- channels for cell motility — The cellular chloride channels CLIC1 and CLIC4 contribute to virus-mediated cell motility — MCPyV ST enhances Cl− channels for cell motility — Supporting Information 

# The cellular chloride channels CLIC1 and CLIC4 contribute to virus-mediated cell motility

## Supporting Information

- Supp Figs 1 and 2 - Supp Figs 1 and 2
