# Supplementary material for: The cellular chloride channels CLIC1 and CLIC4 contribute to virus-mediated cell motility
Source: J Biol Chem. 2018 Feb 8;293(12):4582–90. doi: 10.1074/jbc.RA117.001343 (PMC5868249; doi:10.1074/jbc.RA117.001343)
Supplement: Supporting Information [file supp_RA117.001343_134501_0_supp_36006_p0phfm.pdf]

The cellular chloride channels CLIC1 and CLIC4 contribute to virus-mediated cell motility

Gabrielė Stakaitytė[1,2#], Nnenna Nwogu[1,2#], Jonathan D. Lippiat[3], G. Eric Blair[1], Krzysztof Poterlowicz[4], James R. Boyne[4], Andrew Macdonald[1,2], Jamel Mankouri[1,2], and Adrian Whitehouse[1,2]

[1]School of Molecular and Cellular Biology, [2]Astbury Centre for Structural Molecular Biology, [3]School of Biomedical Sciences, Faculty of Biological Sciences, University of Leeds, Leeds, LS2 9JT, [4]Centre for Skin Sciences, School of Chemistry and Biosciences, Faculty of Life Sciences, University of Bradford, Bradford, BD7 1DP, United Kingdom

Supplementary Information

Contents:

Supplemental Figures (Figures 1-2)

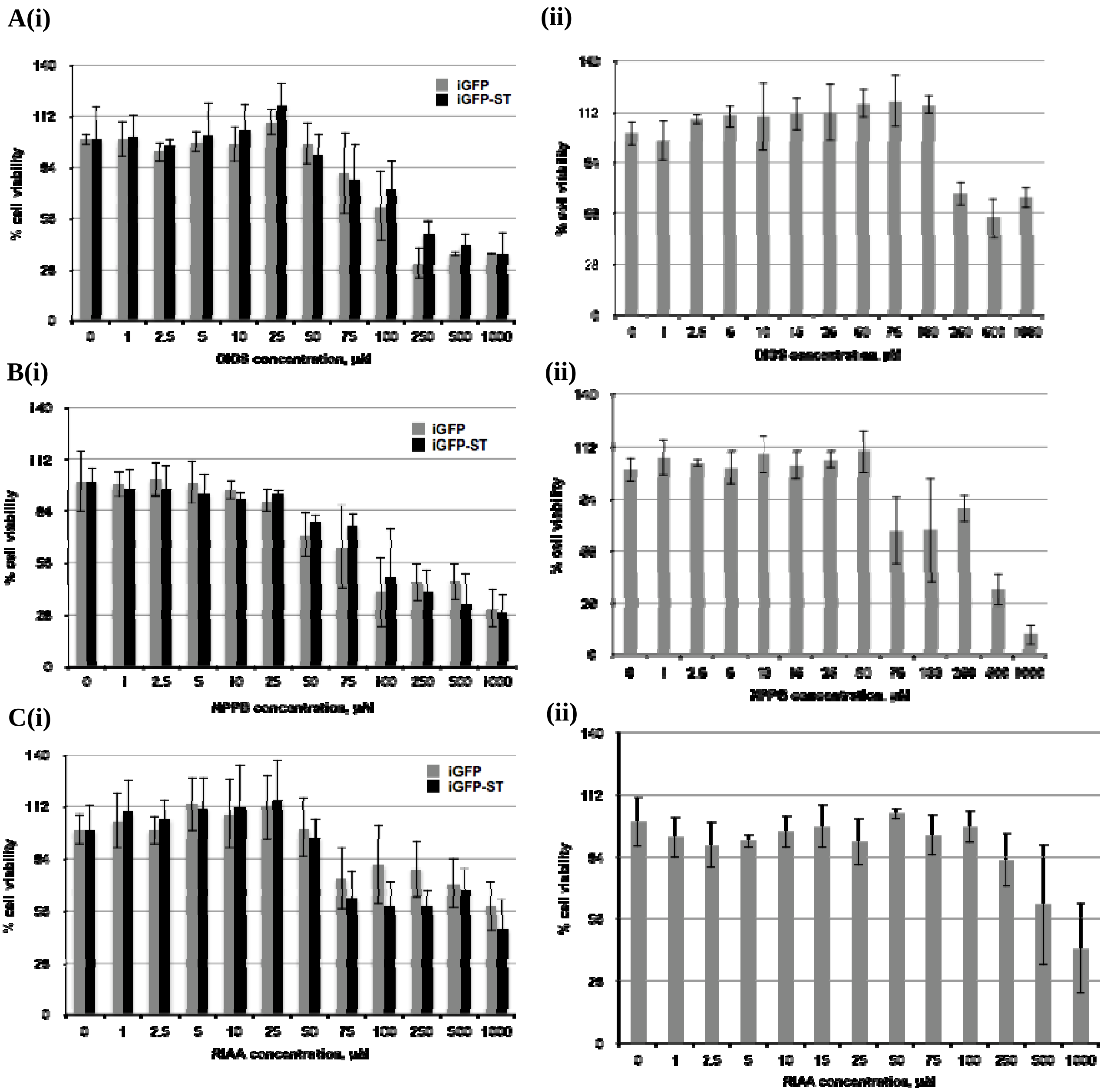

Supplementary Figure 1. Selection of non-toxic concentrations of each Cl- channel modulator. Cell viability was measured in **(A)** i293-EGFP and i293-EGFP-ST and **(B)** MCC13 cells using an MTS-based assay in the presence of increasing concentrations of **(i)** DIDS, **(ii)** NPPB and **(iii)** RIAA inhibitors.

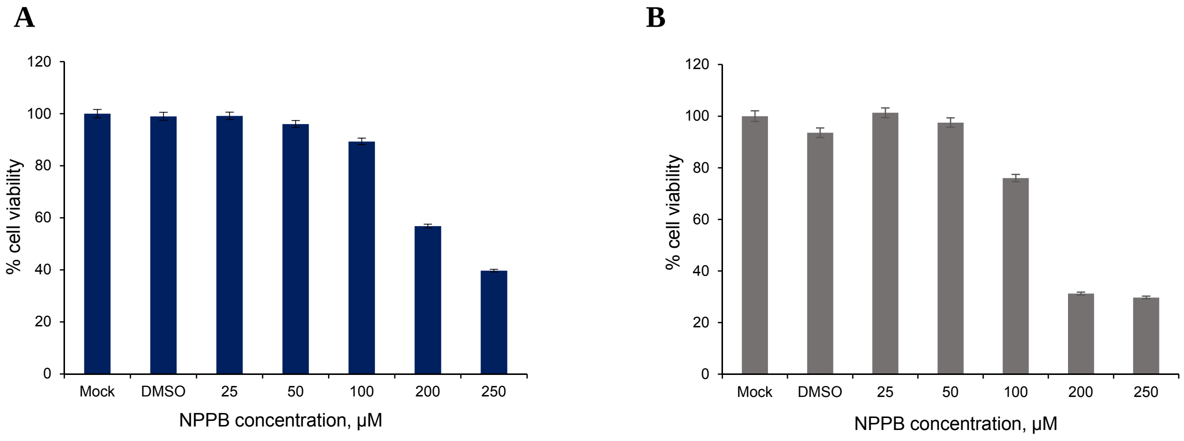

Supplementary Figure 2. Selection of non-toxic concentrations of NPPB channel modulator in MCPyV positive Merkel cell carcinoma cell lines. Cell viability was measured in **(A)** PeTa and **(B)** Waga cells using an MTS-based assay in the presence of increasing concentrations of NPPB inhibitor.
